# Telemedicine remote controlled stroke evaluation and treatment, the experience of radiographers, paramedics and junior doctors in a novel rural stroke management team

**DOI:** 10.1186/s12913-021-06591-1

**Published:** 2021-06-05

**Authors:** Elin Kjelle, Aud Mette Myklebust

**Affiliations:** Department of Optometry, Radiography and Lighting Design, Faculty of Health and Social Sciences, University of South-Eastern Norway, University of South-Eastern Norway, Post office box 235, 3603 Kongsberg, Norway

**Keywords:** Rural, CT, Telemedicine, Stroke management

## Abstract

**Background:**

In the case of ischemic stroke, access to a Computed Tomography (CT) scanner and the start of thrombolytic therapy immediately is imperative. Transport to the nearest hospital from the remote, rural area of Hallingdal in Norway entails a 2–3 h drive. The local medical centre in this area has a CT-scanner operated by radiographers during office hours. Out-of-hours stroke evaluation and treatment has been the focus of a research project. Paramedics position the patient in the CT-scanner. A radiographer at the participating hospital runs a remote-controlled scan and a junior doctor instructs a paramedic in the performance of a neurological examination. The aim of this study was to explore how radiographers, paramedics and junior doctors experience conducting telemedicine-based stroke evaluation and treatment.

**Methods:**

Six semi-structured interviews were conducted with radiographers, paramedics and junior doctors; with remote control, CT examination and stroke management as central issues. Core issues in the interview guide were: communication; cooperation; competence; service quality and training. The study employed thematic content analysis in analysing the data inductively.

**Results:**

The analysis gave an overview of the patient flow and communication routines in this service. Further findings were divided into two main themes, “Teamwork” and “Quality”. The theme “Teamwork” included three categories “Communication”, “Trust and confidence”, and “Task and task shifting”. The theme “Quality” included two categories “Education and training” and “Safety and routines”. The respondents considered the service to be of high quality and that the team functioned at a high level as a result of regular training sessions. However, communication and image reading routines could be improved.

**Conclusions:**

The telemedicine-based, remote controlled, stroke evaluation and treatment was experienced, by the participants, to be well organised and of high quality. Communication and image reading appear to be the salient challenges. Regular training sessions and follow-up, as well as an evaluation of incidents by the project manager, proved to be of great importance in retaining and securing the continued running of the service and ensuring high-quality treatment. Further research is indicated in the comparison of this telemedicine service with stroke treatment given in a mainstream hospital.

## Background

To distinguish between acute ischemic stroke and intracerebral haemorrhage an acute non-contrast CT examination is the preferred diagnostic tool [1–3]. In the case of an ischemic stroke, starting thrombolytic therapy (Alteplase© treatment) as soon as possible and at most 4.5 h after the onset of symptoms, will reduce morbidity and mortality in stroke patients [1, 3]. The effect of Alteplase treatment decreases every 15 min [3]; thus, fast access to a CT scanner and a stroke team to administer Alteplase is critical for stroke patients [2, 3]. For patients living in rural areas, the travel time to hospital is usually too long to be able to provide Alteplase treatment within the critical timeframe [4]. From the Hallingdal area of Norway there is a 2-3-hour drive to the local hospital [4]. The local medical centre in this area installed a CT scanner in 2016, with radiographers operating the scanner during the daytime, Monday-Friday [4]. A video link to the local hospital has been installed providing stroke treatment to patients by means of a stroke team consisting of personnel from both the local medical centre and the hospital as a telemedicine-based service [4]. However, the absence of a physician and radiographer out-of-hours precludes a full-time service, and thus the offer of equal access to the service for stroke patients in urban areas [4].

Ibsen and Hall [4] initiated a project in 2019 to keep the service open outside office hours using available on-call staff, this project is on-going and data collection will end in 2021. In this project, the paramedics position the patient in the CT scanner guided by a radiographer at the local hospital via video link. The radiographer at the hospital then runs the scan remotely [4]. Using the video link, a junior doctor at the local hospital observes and guides the paramedic in the performance of a neurological examination (National Institute of Health Stroke Scale (NIHSS)) and the junior doctor assess the NIHSS score and CT images to decide whether to administer Alteplase or not. When appropriate the paramedics administer Alteplase in the scanner room guided by the junior doctor. The patient is then transferred, with the Alteplase infusion in place, to either the local or tertiary hospital, dependent upon the size and position of the thrombus [4].

Training and regular maintenance for the paramedic is essential to perform correct patient positioning, NIHSS and Alterplase treatment; the project manager (a specialist in internal medicine) provides this training. When non-radiographic professionals operate radiographic equipment and position patients for examination, including CT, it is important to ensure a high-quality, customised training program to ensure a good quality examination [5, 6]; in this project, a radiographer gives this training. Further, both the radiographer and junior doctor are required to communicate and co-operate efficiently with the paramedics via video conference in the same way as the hospital stroke team, in order to efficiently diagnose and begin the treatment of a stroke with Alteplase without delay [1–3, 7].

The establishment of a novel approach to stroke team organisation necessitates an evaluation of the effects of the service. Accordingly, the objective of this study was to explore the experiences of radiographers’, paramedics’, and junior doctors’ in relation to the quality of telemedicine-based stroke evaluation and treatment including a remote controlled CT scan. This paper is the first of two papers evaluating a tele-medical stroke evaluation team.

## Method

This is a qualitative study based on semi-structured interviews. Before describing the methods in detail, the study context is presented.

### *Context: The Norwegian healthcare system and rural tele medical stroke evaluation and treatment in Hallingdal*

The Norwegian healthcare system is a largely public system based on general taxation. The system is managed politically at ministerial and municipal levels [8]. Specialised healthcare, including imaging services, is principally provided by hospital trusts [8]. The Co-ordination Reform, implemented in Norway in 2012 aimed to improve public health and the quality of health services in a sustainable manner, as well as improving the proximity of services to patients in rural areas [9]. Accordingly, local medical centres (community hospitals) organised under the larger hospitals were established to provide health services for the local population and visitors as a combination of municipal and hospital care, a form of 1.5 level healthcare [10, 11]. In some of these centres, as in Ål in Hallingdal, there is an X-ray machine and a CT scanner [11]. At the ambulance station at Ål there are 25 paramedics. In the Hallingdal area, about 50 people suffer a stroke yearly. To date, circa 110 patients (about 40 remote controlled) have been scanned at the local medical centre, 30 % were given Alteplase.

### Participants

Semi-structured interviews were conducted with six health professionals involved in the remote CT service, considered an acceptable sample size for studies that utilise a qualitative approach using content analysis [12]. The participants were recruited from the local hospital and pre-hospital service using volunteer sampling [13]. All participants volunteered by contacting researcher EK after an invitation was sent to the hospital trust defining the inclusion criteria. The inclusion criteria were, radiographers, paramedics, or physicians who had been involved in performing remote CT-scan within the last 3 months. Participants received an information letter and consent form via e-mail. Participants returned a signed consent form before the interview commenced. Two radiographers, two paramedics and two physicians participated in this study.

### Data collection

The semi-structured approach was chosen to ensure that the same topics were discussed with all participants, whilst allowing relevant topics to be explored as they arose [14]. An interview guide, available in Table 1, with open-ended questions was developed by the authors. Core issues included for discussion was; competence, training, communication, cooperation and the quality of the service. The interviews were conducted in June 2020, and were carried out online, due to the Covid-19 pandemic, using the Microsoft Skype for Business (2016) software. A Zoom H1 Handy Recorder recorded the dialogue of the interviews. EK and AMM interviewed all participants jointly and took notes. The interviews lasted on average 33 min (22–38 min). In order to reach consensus [12], at the end of each interview the interviewer summarised the participant’s statements of the main subjects in the interview. The authors are both female radiographers with more than ten years of clinical experience; now working as researchers. Both themselves and the project was presented the participants as an introduction to the interview.

**Table 1:** The interview guide

#	Question
1	What is your experience in working with remote controlled CT stroke evaluation?
2	What kind of training and education did you receive?
3	Do you feel secure with your tasks in the team?
4	Please tell us about how this service is organised.
5	Why do you think this service is important for the Hallingdal area?
6	How would you rate the quality of this service and how is it functioning?
7	What challenges are there in the delivery of this service?

### Ethics

The Norwegian Centre for Research Data approved the handling and storage of personal information in this study (Ref. 358,427). Formal ethics approval is not required according to “Lov om medisinsk og helsefaglig forskning (helseforskningsloven)” https://lovdata.no/lov/2008-06-20-44.

### Analysis

Inductive thematic content analysis was employed to analyse the data. Content analysis is a well-established approach in thematic analysis of semi-structured interviews [12]. The analysis process consisted of six steps; transcription, familiarisation, coding, condensation, categorisation and themes through interpretation based on the descriptions of content analysis of Graneheim and Lundmann [15]. A description of the steps used in this analysis is shown in Table 2.

**Table 2:** Description of the steps of thematic content analysis used in this study

Step	Action
Transcription	The interviews were transcribed verbatim by EK.
Familiarisation	Both authors read the transcripts to get an overview of the content.
Coding	Both authors coded all the transcripts, and met to compare codes and discuss the codes used.
Condensation	The meaning units were sorted by codes in an Excel spreadsheet. Meaning units were colour coded per respondent. Both authors condensed 50 % of the meaning units and marked important quotes.
Categorisation	Through discussion, condensed meaning units were sorted into categories and sub-categories.
Themes	Through discussion, categories were sorted into themes.

## Results

**Fig. 1:**
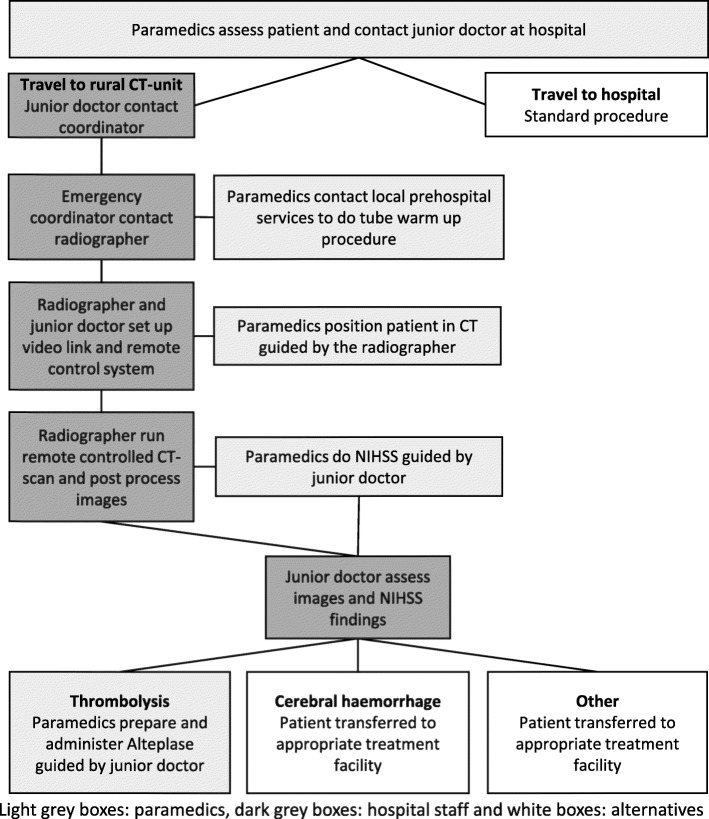
An illustration of the procedure for tele-medical stroke evaluation and treatment in Hallingdal

The steps in the process of telemedicine stroke evaluation and treatment, as described by the respondents, are illustrated in Fig. 1. Paramedics reach the patient, if a stroke is suspected they contact the junior doctor on call at the hospital. The junior doctor decides whether to use the tele-medical service, based on travel distance and the patient’s symptoms. In the hospital, the junior doctor contacts the co-ordinator in the emergency room who in turn contacts the on-call radiographer. The ambulance drives towards the local medical centre and requests another paramedic team to start the CT warm-up procedure. The radiographer and junior doctor prepare the video link and remote control systems. Arriving at the local medical centre, the paramedics position the patient guided by the radiographer. The scan is executed and the radiographer transfers the images. The paramedics perform the NIHSS guided by the junior doctor. The junior doctor evaluates the NIHSS findings and the CT-images and a decision on Alteplase administration and/or the subsequent transfer location is reached.

**Fig. 2:**
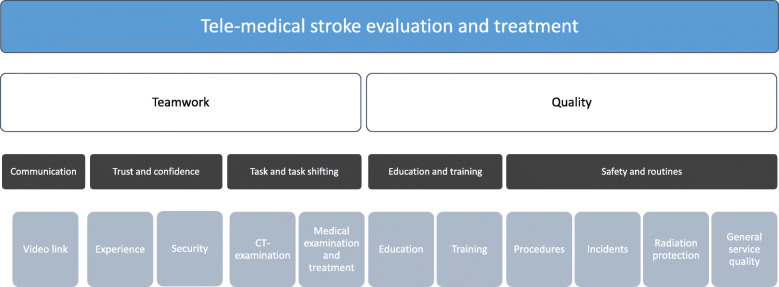
Presentation of the main themes (white boxes) with associated categories (dark grey boxes) and sub-categories (light grey boxes)

To analyse the professionals’ experience of working with and of the quality of this service, condensed text from the transcribed interviews were allocated to five categories with one to four sub-categories. These were further organised into two main themes, equality important for the delivery of the tele-medical stroke evaluation and treatment. Figure 2 presents the main themes with associated categories, and sub-categories. Theme 1, “Teamwork”, included three categories; “Communication”, “Trust and confidence”, and “Task and task shifting”. Theme 2, “Quality” included two categories, “Education and training”, and “Safety and routines”. These themes and categories will be described in detail and illustrated below using key quotes from the respondents.

### Theme 1: Teamwork

#### Communication

 The participants generally describe communication to be good. They are able to see and hear each other and have developed routines to avoid crosstalk by restricting the number of people in the scanner room. Regardless, miscommunication sometimes occurred or there was difficulty in understanding each other, especially during the NIHSS procedure. The junior doctors experienced that paramedics and doctors use slightly different terminology, sometimes making the communication of the patient’s condition or symptoms difficult. One of the junior doctors described a situation where a local physician, who by coincidence was at the medical centre when the patient arrived, stepped in to help. This made communication regarding the patient and the findings of the neurological examination easier. One junior doctor said:

“*I feel we have a slightly different language somehow, that we [paramedic and junior doctor] communicate in a slightly different way. … When the physician was in the room, the communication was much easier. He was a big help in that situatio*”.

The radiographers and paramedics experienced good communication. The radiographers observed a development in skill and understanding in the paramedics through the project. One radiographer said:

*“It is a good communication. It is very good actually. They [paramedics] have more experience now, so that they understand more how things actually work”.*

#### Video link

During the scan and treatment of the patient, the staff communicate using both the video link system and by telephone. In the scanner room, there is a video communication system where the radiographer and the junior doctor can communicate with the paramedics. In this way, they are able to see as well as hear everything going on in the room. In the control room, the paramedics and the radiographer communicate by telephone to perform the scanning. The video communication helps the radiographers to check the patient positioning and isocentre placement. The radiographers experience that seeing the patient positioning before starting the scan is essential for performing a good quality scan. The radiographers are uncertain as to whether it would be possible to have such a service without being able to check patient positioning before the scan. One radiographer said:

“*The video is essential when it comes to patient positioning*”.

The junior doctors find the video essential as well. It is important for them to be able to observe the patient from arrival to the end of the scan and into the NIHSS examination. Observing the patient provides a lot of information; but does not completely compare to being in the same room. They are not able to touch and feel the patient to check muscle tonus and it is hard to observe the patient’s eye movements. Still, they experience that the paramedics know what to do and that the communication is adequate. One junior doctor said:

“*The paramedics are good, they know what to do, but I cannot see what they see. There is an extra link between the patient and me, and I’m supposed to interpret the findings*”

The paramedics experience the video communication as an observation system that works as a support and safety measure. They experience the junior doctor and the radiographer watching them and communicating with them as a good thing, and a help in a sometimes hectic and stressful situation. One paramedic said:

*“From the moment we open the door to the scanner room they watch us all the time. If we do anything wrong, they [radiographer and junior doctor] tell us right away”.*

### Trust and confidence

#### Experience

The participants all experienced that their competence had grown during training and when experiencing live cases. Both junior doctors and radiographers experienced that the paramedics increased their level of competence and their ability to perform allocated tasks throughout the project. The paramedics themselves felt more confident after having experienced the real thing a few times. However, the scarcity of cases precluded confidence in performing the tasks alone. One paramedic said:

*“We will never be fully comfortable with this [stroke treatment]. Nevertheless, as we keep training regularly and the fact that they [project management] follow up, makes it feel safe.”*

#### Security

The participants mainly trusted the other professionals to perform their tasks and included them in the co-operation during the patient situations. Moreover, they felt that the situation was under control as long as everybody remained calm and focussed. The junior doctors trusted the paramedics in the performance of the NIHSS but were not sure if they would be comfortable starting the Alteplase without seeing the patient on video. When asked what they would do if the video was down one junior doctor said:

“*Good question, if I would have dared, hm… I think maybe that I would have used FaceTime on my smart phone".*

The paramedics and radiographers trusted the junior doctors to decide how and where to treat the patient. The junior doctors however, were doubtful. They experienced the telemedicine situation to be stressful as they were already insecure as junior doctors. One junior doctor said:

“*I think I was generally insecure in the beginning. All the medical procedures were new and then this [telemedicine] was something I had never done before. That made it even scarier*”.

### Task and task shifting

#### The CT-examination

The paramedics were trained to independently run a tube warm-up procedure on the CT-scanner. However, patient positioning and scanning was supervised or remotely controlled by a radiographer. There was a discussion regarding task shifting when planning the project. The radiographers were dubious when the project suggested that paramedics should perform the whole scan independently, both unenhanced and a contrast enhanced CT Angiography (CTA) of the brain. Through discussion, the radiographers at the medical centre and the project radiographer developed a procedure whereby radiographers guided the paramedics in positioning and then remotely controlled an unenhanced scan. After the first year of the project, the radiographers trusted the paramedics to position the patient alone, should the video link be down, as long as they had done the positioning on real patients a few times before. However, the radiographers were concerned with the possibility of mistakes causing unnecessary radiation dose to the patient. One radiographer said:

*“We [radiographers] were sceptical to giving up our responsibilities in the beginning. But now we are doing the actual scan and are still responsible for the scanning”*.

Despite the radiographers’ misgivings, the paramedics felt welcome in the project, and the radiographers working daytime in the local medical centre were helpful in training them. The radiographers welcomed them in observing patient scans during the dayshift, outside scheduled training sessions. The paramedics felt excited about being part of the project and were proud to be trusted with these new tasks. They also felt safe with the project manager and felt it was possible to ask any question. One paramedic said:

“*It is fun being part of this project, the project manager has been great. He was always calm, no matter how stupid our questions were*”.

##### Medical examination and treatment

Performing the NIHSS is a central part of the diagnostics to decide whether to perform the Alteplase injection or not. All participants described this as an important part of the service. The paramedics found this task to be interesting and, through training, relatively easy to perform. The junior doctors on the other hand found the NIHSS to be difficult to interpret when not in the same room as the patient; especially if the patient’s symptoms were uncharacteristic of stroke, or if there were other significant neurological findings than those covered by the standard NIHSS. One junior doctor said:

“*It is difficult examining the patient via telemedicine, to do an exhaustive NIHSS. … It is harder with the difficult patients and for them [paramedics] to describe more about their findings outside of the NIHSS*”

Reading CT-images was an undertaking left to the junior doctors. The radiographers considered it an easy task for the junior doctors to perform. The junior doctors on the other hand found reading CT images difficult. The junior doctors would have liked a radiologist to read the CT. One junior doctor said:

“*I think a radiologist should be involved or at least we could send the images to another hospital for a radiologist to read them*”.

To weigh up for the lack of a radiologist the junior doctors needed support from a consultant physician or the project manager to help interpret the images. The junior doctors leaned heavily upon the project manager and could call him at any time. This seems to be imperative for the junior doctors to feel safe about the decision to start the Alteplase treatment or not. One junior doctor said:

“*The project manager has been involved in all the cases. He has participated via FaceTime, looking at the images with me. It makes me feel safer about my medical decisions*”.

In the cases where patients were to be given Alteplase, the paramedics prepare the bolus injection and the infusion in the scanner room guided by the junior doctor. They used the video for the junior doctor to check that the dosage is correct before the bolus injection, and further for checking the mixing of the infusion and the infusion rate. The paramedics found thrombolysis to be an exciting new task and found the possibility for double-checking by the junior doctor to be a good thing. One paramedic said:

“*We prepare an infusion with a drop count of 80. The doctor can zoom in with the camera, and when we both are happy with the drop count we start the transfer of the patient*”.

### Theme 2: Quality

#### Education and training

##### Education

In order to secure good quality treatment and care in the remote controlled service, education and training was required for all personnel groups. The paramedics received lectures from the project manager and others from the hospital in brain anatomy, physiology, and stroke. Further, they were certified in performing the NIHSS, and trained in radiation protection. Radiographers at the local medical centre and the project radiographer at the hospital gave instruction in tube warm-up procedure, patient positioning and the performance of a remote controlled CT-scan. The paramedics thought the lessons were good and necessary for them conduct their tasks in the project. One paramedic said:

“*We had on-site and video lectures on anatomy, physiology and stroke. In addition, a little bit about NIHSS. … We also did a course in radiation protection*”.

### Training

The project leader arranged weekly training sessions. Training with one of the paramedics as a patient for the positioning and NIHSS evaluation, and scanning of a melon or a cabbage as a phantom. Additionally, the paramedics trained in preparing the Alteplase injection and infusion. The paramedics joined the sessions on a rotational basis, with all paramedics participating in the training at least once a month. The project radiographer organised a rotation for the radiographers to alternate in participating in the training sessions. The junior doctors’ inclusion was more infrequent but included at least one training session before going on call. Both the radiographers and junior doctors described it as an individual duty to stay up to date with procedures and update their knowledge if required. One radiographer said:

“*It is your own responsibility to stay up to date with procedures*”.

All groups rated the practical training sessions as good, structured and essential to the smooth running of the service. Repeat training was considered important because the number of patients per person was low. The junior doctors did not have the time to join sessions very often but they were necessary to obtain an overview of what to do, and how to communicate with the rest of the team. The radiographers needed, for the most part, the training session to learn how to guide others in patient positioning. The role as a supervisor was new for the radiographers. One radiographer said:

“*We radiographers do not usually play the role of a supervisor. However, we get good practice in the training sessions*”.

#### Safety and routines

##### Procedures

The radiographers use the same scan procedure as in the hospital to ensure comparable image quality. The CT-scanner in the local medical centre is similar to the hospital’s previous equipment and consequently, radiographers were familiar with the equipment and software at installation. The present CT equipment at the hospital is now from another vendor, causing problems for the radiographers.

The project has developed several procedures specifically for the paramedics to follow during stoke evaluation and treatment. Both physicians and radiographers were involved in developing the procedures for the CT-scanner, the NIHSS procedure and for medication preparation and administration. The paramedics are used to working based on procedures. One paramedic said:

“*We have a folder with procedures which we can follow, with images and everything. You cannot do anything wrong if you follow the guidelines*”.

The junior doctors do not regard the procedures as positively as the other professionals do. They describe the administration of Alteplase, outside of the hospital, as a calculated risk. The drug has side effects and usually doctors in the hospital would tackle them. With the remote service, the complications could develop during transfer, and need to be handled by the paramedics. One junior doctor said:

“*The patient developed complications during transfer. However, this is a calculated risk you take*”.

##### Incidents

The participants described different incidents encountered during training and in real life situations. They were especially concerned with technical failure of the video link or remote control network. They experienced a flexibility and a willingness in the team to improvise when things did not go as planned. The project manager was an important factor in this, in addition to the local system for reporting and learning from incidents. One radiographer said:

“*These things are registered in the report system, and you would tell the project manager*”.

Moreover, both junior doctors and radiographers were clear that if anything should go wrong with the treatment, based on lack of training, it would be a system failure. There have been concerns raised in training and incidents reported, leading to the view that management should do something to improve the situation. One junior doctor said:

*“I think that if anything should go wrong it is the system failing. Because we have told them we feel inadequate to read the CT images”.*

If the video system failed, FaceTime on an iPad from the ambulance and/or private smart phones was used to communicate and observe the patient. The paramedics have an emergency procedure in the CT control room to use if the remote control function should fail while a patient is in the scanner. They felt comfortable using this procedure and preforming a scan supervised only by telephone. The radiographers, on the other hand, did not know about this emergency procedure and said the paramedics could not perform a scan without the remote control functioning. On radiographer said:

“*That [scanning] is outside the paramedics’ mandate, you know, beyond the standard procedure*”.

##### Radiation protection

The radiographers regarded radiation protection for patient and personnel as their responsibility, just as in any ordinary scan. They also felt in control because they were in charge of the scan parameters, and could check if everybody had left the room or wore protective clothing when in the scanner room. One radiographer said:

“*Radiation protection, yes I feel we [radiographers] control the situation*”.

The paramedics experienced the situation as safe. They always assessed the patient before leaving the scanner room; deciding if he/she would be able to lay still or if one of them should remain in the room during the scan, wearing a lead apron and thyroid protection. They had learnt where to stand in the scanner room to reduce radiation exposure and had protective gear easily available. When in doubt the paramedic dressed in lead protection in readiness to go in quickly if the patient started moving around. On paramedic said:

“*Yes, if the patient is a bit restless we [paramedics] try to be prepared, dressing in protective gear*”.

##### General quality of the service

Overall, respondents experienced the service to be of high quality and a good alternative for patients living far from the hospital, or for tourists. The experience of working for an important project was inspiring for all the respondents. The possibility to start the treatment up to three hours earlier was considered an enormous benefit in avoiding severe complications after a stroke. Moreover, as long as the video and network were functioning, none of the respondents doubted that this service is equal to hospital treatment. One junior doctor said:

“*It is a good service, making it possible to do a thrombolysis fast, it works very well*”.

## Discussion

All participants found the project interesting and exciting to be involved in. They considered the service to be of good quality. The use of disparate terminology in communication between doctors and paramedics was challenging. This was especially true during the performance of the NIHSS, or in reporting symptoms. Earlier research has shown that different professions in health-care have their own linguistic style and that the difference in power and authority affects interdisciplinary communication [16, 17]. The paramedics described the junior doctors as having a good way of describing how to perform the NIHSS through training, and that they all said the same thing every time. This made it easier for the paramedics to perform their tasks. The communication of symptoms remained a challenge. In part this could be attributed to the complexity of the symptoms and the difficulty in describing them outside the NIHSS without both professions knowing and using the appropriate medical terms.

### Security and privacy

The video link and CT-scanner remote control were considered essential for the service to be safe for the patient. The team managed to overcome a lack of video link using other devices to communicate. They used the ambulance iPad and private smart phones to communicate, allowing the junior doctor and the radiographer to observe the patient and check their positioning in the scanner. In the video link system privacy and security is protected by the Norwegian Health network, which is used by hospitals and other health care providers in Norway [18]. The use of private smart phones is problematic with regard to patient security and privacy [19]. The Code of Conduct for information security and data protection in the healthcare and care services in Norway [19] states; health care personnel’s private phones should not be used in patient consultations, because of the lack of data encryption. According to fact sheet 54 of the code of conduct for information security and data protection, the use of private smart phones can, in critical situations, be defended in individual cases, nonetheless it should not develop as a systematic practice [20]. To use private phones in an emergency with a patient if the video link fails during the procedure would seem to be within the regulations, as long as the video link is the main system for regular communication in the service. Notwithstanding, the junior doctors seem to use their private smart phones on a regular basis to consult the project manager. This would appear to be at odds with the regulations from the directorate of eHealth [19].

Radiographers considered the loss of connection to remote control of the CT-scanner a major issue. They were not aware of an emergency protocol the paramedics could use to perform the scan if the remote control system was down, and the radiographers considered the paramedics unqualified in performing the scan with only telephone supervision. The paramedics knew about the guideline and were confident they would be able to do the scan unsupervised. The project should address this issue. A consensus on this matter would help secure a safe service for patients.

### Task shifting

In this service, the different professionals were allocated unfamiliar tasks or roles. Notably the paramedics, as both radiographer and physician tasks were shifted to this group of professionals. The paramedics found this exciting and felt secure in the tasks they were set to perform. They ascribed this to the extensive and continuing training, clear guidelines and the guidance from the junior doctors and radiographers in live cases. This concurs with Joshi et al’s review findings (21), that training and good procedures are main enablers for a successful task shifting intervention. The junior doctors and radiographers on the other hand were give a new role in this service. The role of supervisor or guide was new to them and felt challenging. More training in supervising others might help the radiographers and junior doctors to feel more confident in their new role.

### Diagnosing the patient

For junior doctors two main challenges arose through this service, the first was observing and evaluating the patients’ symptoms remotely when guiding the paramedics in the NIHSS evaluation. As discussed by Bagot et al. [7], a major challenge for physicians is the loss of patient cues when not in a face-to-face session, also learning to trust the skills of remote staff. This was a difficult area to address in training sessions and thus created uncertainty and insecurity among the junior doctors. According to Bagot et al. [7]; to feel secure in the new role of telemedicine, physicians need more than 12 months experience. Accordingly, those junior doctors not working with telemedicine on a daily basis would probably need more cases to feel safe in their new role; a fact that may also apply to the radiographers.

The second challenge was interpreting head CT images. Junior doctors and non-radiologists often find this to be a challenging task [22–24], and there seems to be a need for further training in reading head CT. Kelly et al. [24] found in their study that junior doctors significantly improved their reading of head CT for fresh intracranial bleeds when collaborating with radiographers. This may be an opportunity to include the radiographers more fully in the project by giving them training in head CT interpretation as well as the junior doctors. This could provide support for the junior doctors in what they experience as a difficult task, without including staff who are not present at the hospital. In addition, images stored in the hospital’s PACS archive are available to on-call radiologists and neurologists at other hospitals within the trust. It should therefore be possible to include other departments and specialists even though they are not present at the same hospital. This might support the junior doctors not only in interpreting the CT scan, but also in choosing the right treatment for the patient in difficult cases.

### Health care quality

This tele-medical stroke evaluation and treatment service in a rural health centre provides a new way of organising health services in able to provide fast stroke evaluation and treatment for patients living far away from a hospital. This service is considered by the respondents to reduce the time between the onset of stroke symptoms and, if applicable, the start of Alteplase treatment and thus an increase in the quality of healthcare in this rural area. With a 2–3 h drive to drive to the nearest hospital, this telemedicine service could help reduce mortality and disability after stroke [3] by reducing the time to initiate treatment for stroke patients. In addition to the personal benefits for each patient’s spared disability and mortality after a stroke, there is a potential for a substantial reduction in societal healthcare costs as each stroke case is calculated to cost the Norwegian society about € 56 200 [25].

### Strength and limitations

Recruitment was based on the hospital and pre-hospital services to find participating volunteers. This may have resulted in persons responding positively to being asked to volunteer, in order to show management in a favourable light. On the other hand personnel especially critical to the service could also be motivated to volunteer in order to let their critical voice be heard through the research rather than to management directly. This research describes a project ongoing in one specific area of Norway. Thus, the transferability to other contexts could be difficult. However, rich, thick descriptions of the context and participants would enable the reader to determine transferability [26]. The results are based on participants’ self-reported data, and no attempts have been made to verify their statements independently.

## Conclusions

In conclusion, the remote stroke evaluation and treatment service consists of a team that generally works well together and that offers a beneficial service for patients. Radiographers were especially dubious at the start-up of the project, but through collaboration and communication, the service was set up in a way that ensured patient safety and radiation protection and support for the project among the radiographers. Frequent training sessions including all personnel types were considered essential for the team to function. In terms of communication, there is room for improvement within the team, especially when performing an evaluation of the patient using the NIHSS procedure. The reading of head CT images constitutes a stress factor for the junior doctors, and should be addressed in this and future projects. Further research is required to compare the remote scanned patients’ outcome to standard hospital treatment in order to evaluate patient safety.

## Data Availability

The datasets generated and analysed during the current study are not publicly available due to participant anonymity issues, but are available from the corresponding author on reasonable request.

## Abbreviations

CT: Computed tomography
CTA: CT angiography
NIHSS: National Institutes of Health Stroke Scale
PACS: Picture archiving and communication system
